# Behaviour of motorcyclists and bicyclists before and after a road safety campaign, China

**DOI:** 10.2471/BLT.24.291524

**Published:** 2024-09-25

**Authors:** Peishan Ning, Ruisha Peng, Huiying Zong, David C Schwebel, Cifu Xie, Jieyi He, Peixia Cheng, Li Li, Zhenzhen Rao, Guoqing Hu

**Affiliations:** a Department of Epidemiology and Health Statistics; Hunan Provincial Key Laboratory of Clinical Epidemiology, Xiangya School of Public Health, Central South University, 172 Tongzipo Road, Changsha, 410013, China.; b Medical Records Management and Statistical Information Center, Xiangya Hospital, Central South University, Changsha, China.; c Department of Psychology, University of Alabama at Birmingham, Birmingham, United States of America.; d Changsha Center for Disease Control and Prevention, Changsha, China.; e Clinical Trials Center, Affiliated Hospital of Guizhou Medical University, Guiyang, China.; f Department of Child, Adolescent and Women's Health, School of Public Health, Capital Medical University, Beijing, China.

## Abstract

**Objective:**

To examine changes in red-light running and distracted riding among motorcyclists and cyclists before and after the 2020 implementation of the One Helmet, One Seatbelt campaign in China.

**Methods:**

We obtained 192 hours of film before (2019) and after (2021) implementation of the campaign in eight road intersections in Changsha. We calculated percentages and ratios of red-light running and distracted riding. To assess the associations between these traffic behaviours and the campaign, we used multivariable logistic regression models to calculate adjusted odds ratios (aOR).

**Findings:**

We filmed 5256 motorcyclists and cyclists in 2019 and 6269 in 2021. Red-light running decreased from 45.1% to 41.5% during this period (ratio: 0.92; 95% confidence interval, CI: 0.88–0.96), while distracted riding increased from 3.5% to 5.0% (ratio: 1.42; 95% CI: 1.19–1.69). After adjusting for covariates, male riders were more likely to run a red light compared to female riders (aOR: 1.28; 95% CI: 1.06–1.55). Red-light running was also more likely among electric bicycle riders (aOR: 1.46; 95% CI: 1.10–1.95) and motorcyclists (aOR: 1.47; 95% CI: 1.13–1.90) compared to traditional cyclists. All types of riders were less prone to run a red light during peak hours than off-peak hours (aOR: 0.85; 95% CI: 0.73–0.99). Distracted riding was more common on weekends compared to weekdays (aOR: 3.01; 95% CI: 2.02–4.49).

**Conclusion:**

China’s national road safety campaign, which focuses on helmet and seatbelt use, was associated with reduced red-light running. Strict enforcement and targeted modifications could improve the campaign's effectiveness.

## Résumé

**Objectif:**

Étudier les changements en matière de franchissement des feux rouges et de distraction chez les motocyclistes et cyclistes avant et après la mise en œuvre, en 2020, de la campagne «One Helmet, One Seatbelt» (Un casque, une ceinture) en Chine.

**Méthodes:**

Nous nous sommes procuré 192 heures de vidéo filmées à huit carrefours routiers à Changsha avant (2019) et après (2021) la mise en œuvre de la campagne. Nous avons ensuite calculé les pourcentages et ratios de franchissement des feux rouges et de cyclistes ou motocyclistes distraits. Pour déterminer le lien entre ces comportements de conduite et la campagne, nous avons employé des modèles de régression logistique multivariée nous permettant de calculer les odds ratios ajustés (ORA).

**Résultats:**

En 2019, 5256 motocyclistes et cyclistes ont été filmés, contre 6269 en 2021. Le franchissement des feux rouges a diminué, passant de 45,1% à 41,5% durant cette période (ratio: 0,92; intervalle de confiance de 95%, IC: 0,88–0,96), tandis que la distraction a augmenté, passant de 3,5% à 5,0% (ratio: 1,42; IC de 95%: 1,19-1,69). Après avoir pris en compte les covariables, les conducteurs étaient davantage susceptibles de franchir un feu rouge que les conductrices (ORA: 1,28; IC de 95%: 1,06-1,55). Ce franchissement était également plus fréquent chez les vélos électriques (ORA: 1,46; IC de 95%: 1,10-1,95) et les motocyclistes (ORA: 1,47; IC de 95%: 1,13–1,90) que chez les cyclistes traditionnels. Tous les types de deux-roues étaient moins enclins à franchir un feu rouge pendant les heures de pointe que pendant les heures creuses (ORA: 0,85; IC de 95%: 0,73-0,99). Enfin, les cyclistes ou motocyclistes distraits étaient plus nombreux le week-end que la semaine (ORA: 3,01; IC de 95%: 2,02-4,49).

**Conclusion:**

La campagne nationale de sécurité routière en Chine, axée sur le port du casque et de la ceinture de sécurité, a entraîné une baisse du nombre de franchissements des feux rouges. Une application stricte des règles ainsi que des modifications ciblées pourraient améliorer l'efficacité de cette campagne.

## Resumen

**Objetivo:**

Examinar los cambios en la conducción distraída y el cruce de semáforos en rojo entre motociclistas y ciclistas antes y después de la implementación en 2020 de la campaña «Un casco, un cinturón de seguridad» en China.

**Métodos:**

Se obtuvieron 192 horas de grabación antes (2019) y después (2021) de la implementación de la campaña en ocho intersecciones viales de Changsha. Se calcularon los porcentajes y las razones de saltarse semáforos en rojo y de conducción distraída. Para evaluar las asociaciones entre estos comportamientos de tráfico y la campaña, se utilizaron modelos de regresión logística multivariable para calcular las razones de probabilidades ajustadas (aOR).

**Resultados:**

Se filmaron 5256 motociclistas y ciclistas en 2019 y 6269 en 2021. Saltarse los semáforos en rojo disminuyó del 45,1% al 41,5% durante este periodo (razón: 0,92; intervalo de confianza del 95%, IC: 0,88-0,96), mientras que la conducción distraída aumentó del 3,5% al 5,0% (razón: 1,42; IC del 95%: 1,19-1,69). Tras ajustar por covariables, los conductores varones tenían más probabilidades de saltarse un semáforo en rojo que las conductoras (aOR: 1,28; IC del 95%: 1,06-1,55). Además, los conductores de bicicletas eléctricas (aOR: 1,46; IC del 95%: 1,10-1,95) y motociclistas (aOR: 1,47; IC del 95%: 1,13-1,90) eran más propensos a saltarse semáforos en rojo que los ciclistas tradicionales. Todos los tipos de conductores eran menos propensos a saltarse un semáforo en rojo durante las horas de máxima afluencia que fuera de ellas (aOR: 0,85; IC del 95%: 0,73-0,99). La conducción distraída fue más frecuente los fines de semana que entre semana (aOR: 3,01; IC del 95%: 2,02-4,49).

**Conclusión:**

La campaña nacional de seguridad vial de China, centrada en el uso del casco y el cinturón de seguridad, se asoció a una reducción de los conductores que se saltan los semáforos en rojo. Una aplicación estricta y modificaciones específicas podrían mejorar la eficiencia de la campaña.

## ملخص

**الغرض:**

فحص التغيرات في سلوك تجاوز الإشارات الحمراء، والقيادة بلا مبالاة، بين راكبي الدراجات النارية والتقليدية قبل وبعد تنفيذ حملة خوذة واحدة وحزام أمان واحد (One Helmet, One Seatbelt)، عام 2020 في الصين.

**الطريقة:**

لقد حصلنا على 192 ساعة من الفيلم المُصوّر لما قبل (2019) وبعد (2021) تنفيذ الحملة في ثمانية تقاطعات للطرق في تشانغشا. قمنا باحتساب النسب المئوية ونسب تجاوز الإشارات الحمراء والركوب بلا مبالاة. لتقييم الارتباطات بين الحملة وهذه السلوكيات المرورية، استخدمنا نماذج التحوّف اللوجستي متعدد المتغيرات لحساب نسب الاحتمالات المعدلة (aOR).

**النتائج:**

قمنا بتصوير عدد 5256 من راكبي الدراجات النارية والتقليدية في عام 2019، وعدد 6269 من راكبي الدراجات في عام 2021. انخفض سلوك تجاوز الإشارات الحمراء من %45.1 إلى %41.5 خلال هذه الفترة (النسبة: 0.92؛ بفاصل ثقة مقداره %95: 0.88 إلى 0.96)، بينما زاد سلوك الركوب بلا مبالاة بنسبة من %3.5 إلى %5.0 (النسبة: 1.42؛ ‏بفاصل ثقة مقداره %95: 1.19 إلى 1.69). وبعد تعديل المتغيرات المشتركة، كان الركاب الذكور أكثر احتمالية لتجاوز الإشارات الحمراء مقارنة بالراكبات الإناث (نسبة الاحتمالات المعدلة: 1.28؛ ‏بفاصل ثقة مقداره %95: 1.06 إلى 1.55). كما كان تجاوز الإشارات الحمراء أكثر احتمالية بين راكبي الدراجات الكهربائية (نسبة الاحتمالات المعدلة: 1.46؛ ‏بفاصل ثقة مقداره %95: 1.10 إلى 1.95) وراكبي الدراجات النارية (نسبة الاحتمالات المعدلة: 1.47؛ ‏بفاصل ثقة مقداره %95: 1.13 إلى 1.90) مقارنة براكبي الدراجات التقليديين. وكان جميع أنواع راكبي الدراجات أقل ميلاً لتجاوز الإشارات الحمراء خلال ساعات الذروة مقارنة بساعات غير الذروة (نسبة الاحتمالات المعدلة: 0.85؛ ‏بفاصل ثقة مقداره %95: 0.73 إلى 0.99). وكان الركوب بلا مبالاة أكثر شيوعًا في عطلات نهاية الأسبوع مقارنة بأيام الأسبوع (نسبة الاحتمالات المعدلة: 3.01؛ ‏بفاصل ثقة مقداره %95: 2.02 إلى 4.49).

**الاستنتاج:**

إن الحملة الوطنية للسلامة على الطرق في الصين، والتي تركز على استخدام الخوذة وحزام الأمان، قد ارتبطت بانخفاض سلوك تجاوز الإشارات الحمراء. ويمكن أن يؤدي التنفيذ الصارم والتعديلات المستهدفة إلى تحسين فعالية الحملة.

## 摘要

**目的:**

研究中国在 2020 年开展“一盔一带”行动前后，摩托车和自行车骑行人员闯红灯和分心骑行行为的变化情况。

**方法:**

我们获得了长沙八个路口在该行动实施前（2019 年）和实施后（2021 年）的 192 个小时的影像资料。我们计算了闯红灯和分心骑行行为所占的百分比和比值。为了评估这些交通行为与所开展行动之间的关联，我们使用多变量逻辑回归模型来计算调整后的比值比 (aOR)。

**结果:**

我们在 2019 年和 2021 年拍摄到的摩托车和自行车骑行人员总数分别为 5,256 名和 6,269 名。在此期间，闯红灯行为的比例从 45.1% 下降到了 41.5%（比值：0.92；95％ 置信区间，CI：0.88-0.96），而分心骑行行为的比例从 3.5% 上升到了 5.0%（比值：1.42；95% CI：1.19-1.69）。进行协变量调整后，我们发现男性骑行人员闯红灯的可能性高于女性骑行人员（aOR：1.28；95% CI：1.06-1.55）。与传统自行车骑行人员相比，电动自行车骑行人员（aOR：1.46；95% CI：1.10–1.95）和摩托车骑行人员（aOR：1.47；95% CI：1.13-1.90）闯红灯的可能性更大。所有类型的骑行人员在高峰时段闯红灯的可能性都低于非高峰时段（aOR：0.85；95% CI：0.73-0.99）。与工作日相比，周末分心骑行行为更为常见（aOR：3.01；95% CI：2.02-4.49）。

**结论:**

中国开展的全国性道路安全守护行动重在提升头盔和安全带的使用率，这有助于减少闯红灯行为。严格执行和有针对性的改进可进一步提高行动的有效性。

## Резюме

**Цель:**

Изучить изменения в показателях проезда на красный свет и отвлеченной езды среди мотоциклистов и велосипедистов до и после введения в 2020 году кампании «Один шлем – один ремень безопасности» в Китае.

**Методы:**

Было получено 192 часа видеозаписей до (2019 г.) и после (2021 г.) проведения кампании на восьми дорожных перекрестках в городе Чанша. Также были подсчитаны проценты и соотношения случаев проезда на красный свет и отвлеченной езды. Чтобы оценить связь между этими видами поведения на дороге и кампанией, использовались модели многомерной логистической регрессии для расчета скорректированного отношения шансов (сОШ).

**Результаты:**

В 2019 году снято 5256 мотоциклистов и велосипедистов, в 2021 году – 6269. Количество проезжающих на красный свет за этот период сократилось с 45,1 до 41,5% (отношение: 0,92; 95%-й ДИ: 0,88–0,96), в то время как количество случаев отвлеченной езды увеличилось с 3,5 до 5,0% (отношение: 1,42; 95%-й ДИ: 1,19–1,69). После корректировки ковариаций мужчины на велосипедах и мотоциклах чаще проезжали на красный свет, чем женщины (сОШ: 1,28; 95%-й ДИ: 1,06–1,55). Проезд на красный свет также был более вероятен среди водителей электровелосипедов (сОШ: 1,46; 95%-й ДИ: 1,10–1,95) и мотоциклистов (сОШ: 1,47; 95%-й ДИ: 1,13–1,90) по сравнению с обычными велосипедистами. Все типы водителей мото- и велотранспорта были менее склонны проезжать на красный свет в часы пик по сравнению с непиковыми часами (сОШ: 0,85; 95%-й ДИ: 0,73–0,99). Отвлеченная езда была более распространена в выходные дни по сравнению с будними (сОШ: 3,01; 95%-й ДИ: 2,02–4,49).

**Вывод:**

Национальная кампания по безопасности дорожного движения в Китае, в рамках которой основное внимание уделяется использованию шлемов и ремней безопасности, была связана с уменьшением количества случаев проезда на красный свет. Повысить эффективность кампании может строгое соблюдение закона и внесение целевых изменений.

## Introduction

Road traffic injuries among motorcyclists and cyclists are a public health concern. In 2019, road traffic crashes killed 237 533 motorcyclists and 66 102 cyclists worldwide.^1^ In the same year, of all 251 111 road traffic fatalities in China, 16.5% (41 357) were motorcyclists and 9.3% (23 256) were cyclists.^1^

To improve road safety, on 1 June 2020, the Chinese government launched a nationwide road safety campaign, called One Helmet, One Seatbelt, to raise the use of helmets among motorcyclists and electric bicycle riders, and seatbelt usage among car drivers and passengers.^2^^,^^3^ The campaign adopted comprehensive strategies, including helmet donations, law enforcement, enhancing national helmet use education and motorist counselling programmes. Nationwide intervention strategies included on-site education, police warnings, publicizing of typical violation cases and imposing fines for non-compliance.^2^^,^^4^^–^^6^ The campaign has been associated with an increase in overall percentage of helmet wearing, from 8.8% (95% confidence interval, CI: 8.0–9.6) to 62.0% (95% CI: 60.8–63.2),^7^ and the government reported that seatbelt use among drivers and passengers increased to over 90.0%.^5^

We designed the present study to evaluate whether the One Helmet component of the campaign was associated with changes in dangerous riding behaviours among motorcyclists and cyclists not directly targeted by the campaign. The Hawthorne effect suggests individuals alter their behaviour when they are aware of being observed.^8^ In the context of traffic safety, road users may broadly engage in safer behaviours if they know they are being monitored for targeted violations such as not using helmets or seatbelts.

We assessed the association of implementation of the One Helmet, One Seatbelt campaign with changes in two non-targeted dangerous riding behaviours among motorcyclists and cyclists – red-light running and distracted riding. We examined two primary research questions. First, did the campaign reduce red-light running and distracted riding among motorcyclists and cyclists? Second, did the campaign’s auxiliary associations on red-light running and distracted riding vary across demographic groups?

## Method

### Study design

We designed a before-and-after video-based observational study to examine the two research questions. This study builds on data from an observational study conducted between 29 June and 17 July 2019, which investigated the epidemiological characteristics of distracted walking among pedestrians before the national One Helmet, One Seatbelt campaign.^7^

### Data source

To reduce bias in the research design, we adopted the strategy used in the pre-campaign observational study^7^ to collect post-campaign data through video-based observations. Both pre- and post-campaign, we conducted field observations using smartphone-based HD cameras (1080p resolution, Redmi Note 3, Xiaomi, Beijing, China) placed at eight four-leg intersections in Changsha, Hunan province, China. We selected intersections by multistage random sampling (available in authors’ online repository)^7^^,^^9^ and we used the same observational sites in both phases of data collection. In short, we first divided Changsha into 412 square-shaped geographic zones (1.9 km × 1.9 km). We defined eligible zones as having at least two four-leg intersections; 261 (63%) zones met this criterion. Then we randomly chose 10 of the eligible zones and subsequently randomly selected two four-leg intersections within each of those 10 zones, resulting in 20 randomly-selected road intersections for observation. Finally, during pilot observations, four of the selected road intersections included at least one crossroad with 10 or more vehicle lanes, which proved too wide to permit valid data coding using our camera system. Another eight intersections had less than 100 motorcyclists and cyclists passing in a 6-hour videotaped segment. We therefore dropped those 12 intersections and retained the remaining eight for inclusion in this study (online repository).^9^ Each video recorded all passing motorcyclists and cyclists and traffic light sequences for 0.5 to 2 hours.

Because traffic behaviours of motorcyclists and cyclists may vary across weekdays versus weekends,^10^ in both research phases we recorded traffic at each selected intersection for two days, one weekday and one weekend day. Using results from pilot observations, we selected the recording time for each selected day as three peak hours (7:30–9:00 and 17:00–18:30 for weekdays; 8:30–10:00 and 17:00–18:30 for weekends); and three off-peak hours (9:00–11:00 and 16:30–17:00 for weekdays; 10:00–12:00 and 16:30–17:00 for weekends).^7^ We used the same hours for both research phases. In total, we recorded 192 hours of videos: 2 rounds of observations (2019 and 2021) × 8 intersections per observation × 2 days per intersection × 6 hours per day.

### Sample size

Based on previous reports,^7^^,^^11^^–^^15^ we hypothesized that the national campaign would reduce red-light running behaviour by 15% and distracted riding among motorcyclists and cyclists by 50%. Assuming *α*: 0.05, *β*: 0.20, an intracluster correlation coefficient: 0.001 and a cluster size of 450, we calculated the required sample size using observed percentages before and after the campaign. The pre-campaign percentages were 22.9% for red-light running^12^ and 3.0% for distracted riding,^13^ and we assumed the post-campaign percentages to be 19.5% and 1.5%, respectively. To detect a change in red-light running, a minimum sample size of 3278 motorcyclists and cyclists per round of observation was required, while a sample size of 2216 was required to evaluate change in distracted riding. 

### Demographic characteristics

Following methods from previous research,^7^ we trained coders to review the videos and document the demographic characteristics of all observed motorcyclists and cyclists, including: (i) sex (male or female); (ii) estimated age group (< 20 years, 20–49 years, or ≥ 50 years); (iii) type of vehicle (traditional bicycle, electric bicycle, motorcycle); and (iv) occupation (professional deliverer or others), identified through observation of the vehicle and/or uniform and apparel of the rider. We also recorded time (peak hour or off-peak hour), day (weekday or weekend) and observation year (2019 or 2021).

### Outcome measures

We defined red-light running as passing through an intersection when the traffic signal on their intended path displayed a red light according to the *Regulations for the implementation of the law of the People's Republic of China on road traffic safety*.^16^ We calculated the percentage of red-light running, by dividing the number of cyclists or motorcyclists who ran a red light by total number of cyclists or motorcyclists observed, then multiplying by 100. 

Following previous studies and recognizing that distraction falls along a continuum rather than a dichotomy,^17^^–^^20^ we defined distracted riding as any behaviours irrelevant to riding that diverted the rider’s attention away from the road while in motion. Distracted riding reduces rider’s awareness of their surroundings and potentially increases crash risk. Based on pilot observations, we predefined distracted riding behaviours as mobile phone use, talking to pedestrians or passengers, eating, drinking or smoking. The coders manually identified them through video review.^17^^,^^18^^,^^20^ We calculated the percentage of distracted riding by dividing the number of distracted cyclists or motorcyclists by total number of cyclists or motorcyclists observed, then multiplying by 100.

### Reliability of video coding

Using SPSS, version 27 (IBM Corporation, Chicago, United States of America), we randomly selected 5% of videos (10 of 192 hours) to conduct reliability checks of coding accuracy. An independent coder reviewed the randomly selected tapes and demonstrated high reliability (average: 93.4%; range: 92.0–95.1) with the primary coder for all study variables (online repository).^9^

### Statistical analysis

We calculated the percentage and 95% CI of the two behaviours using a binomial distribution. We tested demographic differences between the study samples collected in 2019 and 2021 using *χ^2^* test. To reflect changes in the two behaviours before and after implementing the campaign, we calculated percentage ratios. To assess and quantify the associations between the campaign and dangerous riding behaviours among motorcyclists and cyclists, we used adjusted odds ratios (aORs) based on multivariable logistic regression, which were adjusted for all covariates.

We performed all statistical analyses using SPSS, version 27. A significance level of *P* < 0.05 in two-tailed tests was considered statistically significant.

### Ethical statement

The ethics committee of Xiangya School of Public Health, Central South University, China (approval no. XYGW-2021–72), which approved the study, exempted the research from informed consent requirements. All recorded data were de-identified and used only for research. 

## Results

We observed a total of 11 525 motorcyclists and cyclists, with 5256 (45.6%) recorded before the campaign and 6269 (54.4%) after the campaign. There was a higher percentage of female riders observed after the campaign, 23.4% (1464) compared with 19.3% (1014) before the campaign. Most riders were estimated to be aged 20–49 years (89.6%; 4710 pre-campaign and 94.3%; 5911 post-campaign). The percentage of people riding electric bicycles increased from 17.3% (909) to 29.8% (1868). Additionally, the percentage of professional delivery riders increased from 11.2% (591) to 14.1% (884), and riders on weekdays grew from 38.3% (2013) to 45.6% (2856; Table 1).

**Table 1:** Characteristics of motorcyclists and cyclists observed in 2019 and 2021, Changsha, China

Variable	No. (%)			*P* ^a^
	Both years (*n* = 11 525)	2019 (*n* = 5 256)	2021 (*n* = 6 269)	
**Sex**				< 0.01
Male	9 047 (78.5)	4 242 (80.7)	4 805 (76.6)	
Female	2 478 (21.5)	1 014 (19.3)	1 464 (23.4)	
**Age, years**				< 0.01
< 20	174 (1.5)	104 (2.0)	70 (1.1)	
20–49	10 621 (92.2)	4 710 (89.6)	5 911 (94.3)	
≥ 50	730 (6.3)	442 (8.4)	288 (4.6)	
**Vehicle type**				< 0.01
Traditional bicycle	1 440 (12.5)	939 (17.9)	501 (8.0)	
Electric bicycle	2 777 (24.1)	909 (17.3)	1 868 (29.8)	
Motorcycle	7 308 (63.4)	3 408 (64.8)	3 900 (62.2)	
**Occupation**				< 0.01
Delivery person	1 475 (12.8)	591 (11.2)	884 (14.1)	
Others	10 050 (87.2)	4 665 (88.8)	5 385 (85.9)	
**Time**				0.13
Peak hour	6 162 (53.5)	2 851 (54.2)	3 311 (52.8)	
Off-peak hour	5 363 (46.5)	2 405 (45.8)	2 958 (47.2)	
**Day**				< 0.01
Weekday	4 869 (42.2)	2 013 (38.3)	2 856 (45.6)	
Weekend	6 656 (57.8)	3 243 (61.7)	3 413 (54.4)	

^a^ *P*-values were used to show differences in rider characteristics before and after the campaign.

### Red-light running

After implementing the national campaign, the overall percentage of red-light running among motorcyclists and cyclists decreased from 45.1% to 41.5% (ratio: 0.92; 95% CI: 0.88–0.96. Subgroup analysis showed significant reductions in red-light running among females (ratio: 0.80; 95% CI: 0.73–0.89); riders aged 20–49 years (ratio: 0.91; 95% CI: 0.87–0.95); riders using traditional bicycles (ratio: 0.73; 95% CI: 0.63–0.85); people who were not delivery professionals (ratio: 0.92; 95% CI: 0.87–0.96); riders during peak hours (ratio: 0.89; 95% CI: 0.84–0.94); and riders on weekdays (ratio: 0.88; 95% CI: 0.82–0.94; Table 2).

**Table 2:** Red-light running among motorcyclists and cyclists before and after implementing a national road safety campaign, Changsha, China, 2019 and 2021

Variable	% (95% CI)		Ratio (95% CI)
	Pre-campaign	Post-campaign	
**Overall**	45.1 (43.8–46.5)	41.5 (40.3–42.7)	0.92 (0.88–0.96)
**Sex**			
Male	45.4 (43.9–47.0)	43.4 (42.0–44.8)	0.95 (0.91–1.00)
Female	43.8 (40.8–46.9)	35.2 (32.8–37.7)	0.80 (0.73–0.89)
**Age, years**			
< 20	30.1 (22.1–39.5)	30.0 (20.5–41.5)	1.00 (0.63–1.58)
20–49	45.6 (44.2–47.1)	41.5 (40.3–42.8)	0.91 (0.87–0.95)
≥ 50	43.0 (38.3–47.8)	43.7 (38.1–49.5)	1.02 (0.86–1.21)
**Vehicle type**			
Traditional bicycle	40.1 (37.0–43.3)	29.3 (25.5–33.5)	0.73 (0.63–0.85)
Electric bicycle	38.5 (35.4–41.7)	35.2 (33.1–37.4)	0.92 (0.83–1.01)
Motorcycle	48.3 (46.6–50.0)	46.0 (44.5–47.6)	0.95 (0.91–1.00)
**Occupation**			
Delivery person	45.4 (41.4–49.5)	42.8 (39.6–46.1)	0.94 (0.84–1.06)
Others	45.1 (43.7–46.5)	41.3 (40.0–42.6)	0.92 (0.87–0.96)
**Time**			
Peak hour	46.0 (44.2–47.8)	40.8 (39.2–42.5)	0.89 (0.84–0.94)
Off-peak hour	44.1 (42.1–46.1)	42.2 (40.4–44.0)	0.96 (0.90–1.02)
**Day**			
Weekday	43.2 (41.0–45.4)	38.1 (36.3–39.9)	0.88 (0.82–0.94)
Weekend	46.2 (44.5–48.0)	44.3 (42.6–46.0)	0.96 (0.91–1.01)

CI: confidence interval.

After adjusting for all covariates, reductions in red-light running were still evident following the national road safety campaign, with varying associations observed across different demographic groups. Male riders were less responsive to the campaign than female riders (aOR for interaction: 1.28; 95% CI: 1.06–1.55). Electric bicycle riders and motorcyclists were also less influenced by the campaign (aOR for interaction: 1.46; 95% CI: 1.10–1.95 and aOR for interaction: 1.47; 95% CI: 1.13–1.90, respectively). Riders during peak hours were more responsive to the campaign than off-peak riders (aOR for interaction: 0.85; 95% CI: 0.73–0.99; Table 3).

**Table 3:** Likelihood of red-light running among motorcyclists and cyclists after implementation of a national road safety campaign, China, 2019 and 2021

Independent variable	aOR (95% CI)
**Intervention**	
Pre-campaign	Reference
Post-campaign	0.69 (0.56–0.86)
**Sex**	
Male	1.00 (0.87–1.16)
Female	Reference
**Age, years**	
< 20 years old	0.68 (0.47–0.98)
20–49 years old	1.01 (0.86–1.18)
≥ 50 years old	Reference
**Vehicle type**	
Traditional bicycle	Reference
Electric bicycle	0.88 (0.73–1.07)
Motorcycle	1.34 (1.14–1.56)
**Occupation**	
Delivery person	0.93 (0.78–1.11)
Others	Reference
**Time**	
Peak hour	1.11 (0.99–1.24)
Off-peak hour	Reference
**Day**	
Weekend	1.13 (1.01–1.27)
Weekday	Reference
**Interaction^a^**	
Intervention × Sex	1.28 (1.06–1.55)
Intervention × Electric bicycle	1.46 (1.10–1.95)
Intervention × Motorcycle	1.47 (1.13–1.90)
Intervention × Occupation	0.87 (0.69–1.10)
Intervention × Time	0.85 (0.73–0.99)
Intervention × Day	1.11 (0.95–1.30)

aOR: adjusted odds ratio; CI: confidence interval.

^a^ Reference group for each interaction is the same as for each covariate.

### Distracted riding

The percentage of motorcyclists and cyclists riding while distracted increased from 3.5% (95% CI: 3.0–4.0) in 2019 to 5.0% (95% CI: 4.5–5.6) in 2021 (ratio: 1.42; 95% CI: 1.19–1.69). Subgroup analysis showed similar increases across most subgroups, with ratios ranging from 1.28 to 2.16, except for weekday riders, where distracted riding decreased following the national campaign (ratio: 0.71; 95% CI: 0.53–0.96). Among riders younger than 20 years or older than 50 years, the increase was not statistically significant (Table 4).

**Table 4:** Distracted riding among motorcyclists and cyclists before and after implementing a national road safety campaign, Changsha, China, 2019 and 2021

Variable	% (95% CI)		Ratio (95% CI)
	Pre-campaign	Post-campaign	
**Overall**	3.5 (3.0–4.0)	5.0 (4.5–5.6)	1.42 (1.19–1.69)
**Sex**			
Male	3.9 (3.3–4.5)	5.3 (4.7–6.0)	1.37 (1.13–1.66)
Female	2.1 (1.4–3.1)	4.0 (3.1–5.1)	1.91 (1.17–3.13)
**Age, years**			
< 20	3.8 (1.5–9.5)	11.4 (5.9–21.0)	2.97 (0.93–9.49)
20–49	3.6 (3.1–4.1)	5.0 (4.5–5.6)	1.40 (1.17–1.69)
≥ 50	2.9 (1.7–5.0)	3.1 (1.7–5.8)	1.06 (0.46–2.45)
**Vehicle type**			
Traditional bicycle	4.4 (3.2–5.9)	5.2 (3.6–7.5)	1.19 (0.74–1.92)
Electric bicycle	2.5 (1.7–3.8)	4.8 (3.9–5.8)	1.88 (1.20–2.96)
Motorcycle	3.6 (3.0–4.2)	5.1 (4.4–5.8)	1.43 (1.15–1.78)
**Occupation**			
Delivery person	5.6 (4.0–7.7)	10.1 (8.3–12.2)	1.80 (1.23–2.65)
Others	3.3 (2.8–3.8)	4.2 (3.7–4.7)	1.28 (1.04–1.56)
**Time**			
Peak hour	3.7 (3.1–4.5)	4.9 (4.2–5.7)	1.31 (1.03–1.66)
Off-peak hour	3.3 (2.6–4.1)	5.1 (4.3–5.9)	1.54 (1.18–2.02)
**Day**			
Weekday	4.2 (3.4–5.2)	3.0 (2.4–3.7)	0.71 (0.53–0.96)
Weekend	3.1 (2.5–3.7)	6.6 (5.9–7.5)	2.16 (1.71–2.72)

CI: confidence interval.

After adjusting for covariates, the implementation of the campaign was not overall associated with distracted riding. However, the results suggest that distracted riding was more common on weekends compared to weekdays following the campaign (aOR for interaction: 3.01; 95% CI: 2.02–4.49; Table 5).

**Table 5:** Likelihood of distracted riding among motorcyclists and cyclists after implementation of a national road safety campaign, China, 2019 and 2021

Independent variable	aOR (95% CI)
**Intervention**	
Pre-campaign	Reference
Post-campaign	1.24 (0.68–2.29)
**Sex**	
Male	1.85 (1.16–2.96)
Female	Reference
**Age, years**	
< 20	2.11 (1.00–4.44)
20–49	1.35 (0.87–2.11)
≥ 50	Reference
**Vehicle type**	
Traditional bicycle	Reference
Electric bicycle	0.62 (0.36–1.06)
Motorcycle	0.77 (0.52–1.13)
**Occupation **	
Delivery person	1.79 (1.20–2.69)
Others	Reference
**Time**	
Peak hour	1.18 (0.87–1.59)
Off-peak hour	Reference
**Day**	
Weekend	0.71 (0.53–0.97)
Weekday	Reference
**Interaction**	
Intervention × Sex	0.60 (0.35–1.06)
Intervention × Electric bicycle	1.38 (0.69–2.78)
Intervention × Motorcycle	0.88 (0.49–1.58)
Intervention × Occupation	1.40 (0.85–2.30)
Intervention × Time	0.84 (0.57–1.23)
Intervention × Day	3.01 (2.02–4.49)

aOR: adjusted odds ratio; CI: confidence interval.

## Discussion

Evaluating how the Chinese road safety campaign influenced two non-targeted dangerous riding behaviours, red-light running and distracted riding, revealed two main findings. First, the campaign was associated with reductions in red-light running among motorcyclists and cyclists, although the associations varied across riders’ sex, vehicle type and time of day. Second, distracted riding showed a slight increase following the campaign, although a notable decrease was observed during weekdays.

As part of the One Helmet, One Seatbelt campaign, motorcyclists and cyclists received more targeted education both offline and online.^5^ They were also exposed to extensive visible enforcement measures nationwide, such as live-streamed field enforcement events and live podcasts as well as offline motorist counselling programmes.^5^^–^^8^ These efforts likely raised safety awareness of motorcyclists and cyclists and made them realize they were being monitored by road traffic police officers and volunteers. Such monitoring may have transferred to broad safety engagement, including reducing red-light running.^8^

Three reasons can explain the varied associations observed between red-light running and different covariates. First, female riders are more likely to adhere to rules and engage in fewer risk-taking behaviours compared to male counterparts.^21^ Second, traditional bicycles lack electric power and generally travel at slower speeds, making them more likely to be intercepted by road traffic police officers if they violate red-light regulations. Third, enforcement of regulations by road traffic police officers tends to be stricter during peak hours, which may lead riders to exercise more caution and avoid red-light running and other dangerous traffic behaviours during these hours, but a greater willingness to violate regulations during off-peak hours.^22^^,^^23^

Despite the significant reduction in red-light running following the campaign, more than 29% of riders still ran red lights, suggesting current road traffic safety measures in China are inadequate to fully prevent red-light violations. Enhancing the enforcement of laws against red-light running could improve safety, as enforcement varies widely across jurisdictions. This variation is partly due to insufficient staffing of police departments, and increasing staffing levels could help address the issue.^24^ Additionally, road police officers may prioritize more dangerous violations presenting, such as drink–driving, speeding and fatigued driving^16^, which can lead to less attention on red-light running by motorcyclists and cyclists.^25^^,^^26^

We were surprised to find that distracted riding increased between the survey years. One possible explanation could be the rapid growth in smartphones possession during the study period, including those smartphones that incorporate sophisticated entertainment and navigation applications.^27^ Another contributing factor could be the increase in professional delivery personnel in China during that period. The percentage of delivery personnel in our sample increased by 2.9 percentage points, and those workers often use smartphones for navigation and customer communication, leading to distraction while driving.^28^

While the overall percentage of distracted riders increased after the implementation of the national campaign, distracted riding during weekdays decreased. This decrease may be due to the stricter police enforcement on weekdays during the national campaign, which likely increased riders’ perception and concern about consequences of being caught for distracted riding.

Our findings have two implications for policy. First, they indicate that the national campaign had an auxiliary effect. Together with previously reported improvements in campaign-targeted traffic behaviours,^7^ the One Helmet, One Seatbelt campaign should be continued in China, with a focus on prioritizing strict enforcement of relevant regulations. More broadly, the findings suggest the Hawthorne effect^8^ might apply in traffic safety environments. Efforts to improve traffic behaviour through monitoring, stopping and warning or fining violators can improve safety not just in targeted behaviours, but also across a wider range of traffic violations.

Second, the heterogenous effects we discovered across subgroups and the lack of a reducing distracted riding indicate that the present policy is inadequate and could be improved. For example, the One Helmet, One Seatbelt campaign could be expanded to directly incorporate other dangerous riding behaviours, including red-light running and distracted riding among motorcyclists and cyclists, as well as common pedestrian violations like crossing against signal lights. Additionally, in line with a safe systems approach, engineering approaches, such as installing cameras to monitor risk behaviours at road intersections, should be implemented. For example, policies could be modified to require electric bicycles to be registered with license plates, allowing enforcement of dangerous riding behaviours through the use of cameras at intersections to record violations and issue fines to riders.^29^^–^^31^ Any efforts to improve national traffic safety campaigns must ensure that financial and personnel resources are available to sustain the campaign nationwide over many years.

Our study had some limitations. First, we conducted the study in a single city and future research should assess whether these findings can be generalized to other locations, including rural areas. Second, we evaluated just two auxiliary effects of the national campaign and only within the first year of its implementation. Additionally, we were unable to analyse the effect of the campaign on crashes due to their low frequency. Future research should consider other associations between the campaign and other traffic safety measures, including legal violations among pedestrians and effects for all road users beyond the first year. Third, we manually coded riders’ sex and age based on their physical appearance, which might have led to inaccurate data. We validate the coding by assessing the quality of manual transcription using data from 305 riders who were simultaneously evaluated through manual video assessments and face-to-face interviews. The analysis showed a high consistency of 99.7% (304/305) for age group and 93.1% (284/305) for sex between the two evaluation methods (online repository).^9^ Finally, to ensure that the smartphone cameras could clearly record traffic at all selected road intersections, we only recorded on sunny days or those with light rain. Of the 192 hours of video, light rain was present for less than one hour. Therefore, we believe that weather conditions did not substantially affect our findings.

In conclusion, the national road safety campaign One Helmet, One Seatbelt was associated with reduced red-light running among motorcyclists and cyclists. To maximize the effect of the national road safety campaign, the Chinese government should continue to implement the campaign and should extend it to include other dangerous traffic behaviours. Enforcement of policies may be particularly important to promote safe behaviour among all road users.

## References

[1] Institute for Health Metrics and Evaluation GBD results [internet]. Seattle: University of Washington; 2024. Available from: https://vizhub.healthdata.org/gbd-results/ [cited 2024 Jul 27].

[2] [Traffic management bureau of the ministry of public security deployed the one helmet, one seatbelt security guard operation] [internet]. Beijing: Central People’s Government of the People’s Republic of China; 2020. Chinese. Available from: https://www.gov.cn/xinwen/2020-04/21/content_5504613.htm [cited 2024 Feb 13].

[3] [Ministry of public security requires the safe promotion of the one helmet, one seatbelt safety protection action and actively cooperate with the market supervision department to strictly investigate the illegal behaviour of the price of safety helmets] [internet]. Beijing: Central People’s Government of the People’s Republic of China; 2020. Chinese. Available from: https://www.gov.cn/xinwen/2020-05/21/content_5513522.htm [cited 2024 Feb 13].

[4] [City public security bureau traffic police brigade one helmet, one seatbelt focus on remediation] [internet]. Shanxi: GaoPing Municipal Government; 2021. Chinese. Available from: http://www.sxgp.gov.cn/xwzx_358/jcdt_363/202104/t20210412_1389943.shtml [cited 2024 Feb 13].

[5] [Usage of helmets and seat belts increased significantly since implementing the one helmet, one seatbelt action] [internet]. Beijing: People’s Public Security Newspaper; 2021. Chinese. Available from: https://www.mps.gov.cn/n2254314/n6409334/c7784638/content.html [cited 2024 Feb 13].

[6] [Public security and traffic authorities in China have focused on persuading food delivery riders to wear safety helmets] [internet]. Beijing: People’s Public Security Newspaper; 2020. Chinese. Available from: https://www.mps.gov.cn/n2253534/n4904351/c7201124/content.html [cited 2024 Feb 13].

[7] Ning P, Zong H, Li L, Cheng P, Schwebel DC, Yang Y, et al. Effectiveness of a helmet promotion campaign, China. Bull World Health Organ. 2022 May 1;100(5):329–36. 10.2471/BLT.22.287914 35521031 PMC9047425

[8] Chen LF, Vander Weg MW, Hofmann DA, Reisinger HS. The Hawthorne Effect in infection prevention and epidemiology. Infect Control Hosp Epidemiol. 2015 Dec;36(12):1444–50. 10.1017/ice.2015.216 26383964

[9] Ning P, Peng R, Zong H, Schwebel DC, Xie C, He J, et al. Behaviour of motorcyclists and bicyclists before and after a road safety campaign, China: supplemental file [online repository]. London: figshare; 2024. 10.6084/m9.figshare.27043726.v1 PMC11500252 39464850

[10] Yan F, Li B, Zhang W, Hu G. Red-light running rates at five intersections by road user in Changsha, China: An observational study. Accid Anal Prev. 2016 Oct;95 Pt B:381–6. 10.1016/j.aap.2015.06.006 26152610

[11] Zhang G, Tan Y, Zhong Q, Hu R. Analysis of traffic crashes caused by motorcyclists running red lights in Guangdong province of China. Int J Environ Res Public Health. 2021 Jan 11;18(2):553. 10.3390/ijerph18020553 33440851 PMC7827622

[12] Guo Y, Li Z, Wu Y, Xu C. Exploring unobserved heterogeneity in bicyclists’ red-light running behaviors at different crossing facilities. Accid Anal Prev. 2018 Jun;115:118–27. 10.1016/j.aap.2018.03.006 29558688

[13] de Waard D, Westerhuis F, Lewis-Evans B. More screen operation than calling: the results of observing cyclists’ behaviour while using mobile phones. Accid Anal Prev. 2015 Mar;76:42–8. 10.1016/j.aap.2015.01.004 25590920

[14] Contini L, El-Basyouny K. Lesson learned from the application of intersection safety devices in Edmonton. Accid Anal Prev. 2016 Sep;94:127–34. 10.1016/j.aap.2016.05.023 27286175

[15] Hill L, Rybar J, Jahns J, Lozano T, Baird S. ‘Just Drive’: an employee-based intervention to reduce distracted driving. J Community Health. 2020 Apr;45(2):370–6. 10.1007/s10900-019-00752-4 31564025

[16] [Regulations for the implementation of the law of the People’s Republic of China on Road Traffic Safety. Pub. L. State Council order No. 405 of the People’s Republic of China Oct 7 2017]. Beijing: People’s Republic of China; 2017. Chinese. Available from: https://www.gov.cn/gongbao/content/2019/content_5468932.htm [cited 2024 Jul 29].

[17] Lym Y, Kim S, Kim KJ. Identifying regions of excess injury risks associated with distracted driving: A case study in Central Ohio, USA. SSM Popul Health. 2022 Nov 18;20:101293. 10.1016/j.ssmph.2022.101293 36438079 PMC9682346

[18] Ethan D, Basch CH, Johnson GD, Hammond R, Chow CM, Varsos V. An analysis of technology-related distracted biking behaviors and helmet use among cyclists in New York city. J Community Health. 2016 Feb;41(1):138–45. 10.1007/s10900-015-0079-0 26323983

[19] Ning P, Xie C, Cheng P, Li L, Schwebel DC, Yang Y, et al. Validity across four common street-crossing distraction indicators to predict pedestrian safety. BMC Public Health. 2024 Jan 20;24(1):241. 10.1186/s12889-024-17756-y 38245693 PMC10799455

[20] Wu J, Xu H. The influence of road familiarity on distracted driving activities and driving operation using naturalistic driving study data. Transp Res, Part F Traffic Psychol Behav. 2018;52:75–85. 10.1016/j.trf.2017.11.018

[21] Esmaeli S, Aghabayk K, Bates L. Willingness and intention to run a red light among motorcyclists. J Safety Res. 2022 Dec;83:66–78. 10.1016/j.jsr.2022.08.005 36481038

[22] Pai CW, Jou RC. Cyclists’ red-light running behaviours: an examination of risk-taking, opportunistic, and law-obeying behaviours. Accid Anal Prev. 2014 Jan;62:191–8. 10.1016/j.aap.2013.09.008 24172086

[23] Bai L, Sze NN. Red light running behavior of bicyclists in urban area: Effects of bicycle type and bicycle group size. Travel Behav Soc. 2020;21:226–34. 10.1016/j.tbs.2020.07.003

[24] Jia K, Fleiter J, King M, Sheehan M, Ma W, Lei J, et al. Alcohol-related driving in China: Countermeasure implications of research conducted in two cities. Accid Anal Prev. 2016 Oct;95 Pt B:343–9. 10.1016/j.aap.2016.01.005 26850753

[25] Zhang L, Liu T, Pan F, Guo T, Liu R. [Analysis of effects of driver factors on road traffic accident indexes.] China Saf Sci J. 2014;24(05):79–84. Chinese. 10.16265/j.cnki.issn1003-3033.2014.05.02

[26] [Measures for the administration of points assigned for road traffic violations. Pub. L. Ministry of Public Security order No. 163 of the People’s Republic of China Dec 17 2021]. Beijing: Ministry of Public Security of China; 2021. Chinese. Available from: https://slj.huainan.gov.cn/group1/M00/0B/73/rB406mHT-Q6AROMhAAH2XjnSMZU990.pdf [cited 2024 Sep 17].

[27] [White paper on the psychology and behavior of post-1995 mobile phone use] [internet]. Beijing: School of Psychology and Cognitive Sciences Peking University; 2019. Chinese. Available from: https://aimg8.dlssyht.cn/u/551001/ueditor/file/276/551001/1590392325990843.pdf [cited 2024 Feb 13].

[28] [Meituan delivery platform delivery specifications] [internet]. Beijing: Meituan Rules Center; 2022. Chinese. Available from: https://rules-center.meituan.com/rules-detail/710 [cited 2024 Feb 13].

[29] Zheng W, Qin L, Ning P, Hu G. Association between registration of electric bike (e-bike) with riders’ dangerous riding behaviors and traffic accidents risk: an online survey. Injury Medicine. 2022;11(4):22–7. [Electronic Edition]

[30] Retting RA, Ferguson SA, Hakkert AS. Effects of red light cameras on violations and crashes: a review of the international literature. Traffic Inj Prev. 2003 Mar;4(1):17–23. 10.1080/15389580309858 14522657

[31] Llau AF, Ahmed NU. The effectiveness of red light cameras in the United States-a literature review. Traffic Inj Prev. 2014;15(6):542–50. 10.1080/15389588.2013.845751 24867566

